# Identification of Genetic Defects in 33 Probands with Stargardt Disease by WES-Based Bioinformatics Gene Panel Analysis

**DOI:** 10.1371/journal.pone.0132635

**Published:** 2015-07-10

**Authors:** Wei Xin, Xueshan Xiao, Shiqiang Li, Xiaoyun Jia, Xiangming Guo, Qingjiong Zhang

**Affiliations:** State Key Laboratory of Ophthalmology, Zhongshan Ophthalmic Center, Sun Yat-sen University, Guangzhou, China; University of Iowa, UNITED STATES

## Abstract

Stargardt disease (STGD) is the most common hereditary macular degeneration in juveniles, with loss of central vision occurring in the first or second decade of life. The aim of this study is to identify the genetic defects in 33 probands with Stargardt disease. Clinical data and genomic DNA were collected from 33 probands from unrelated families with STGD. Variants in coding genes were initially screened by whole exome sequencing. Candidate variants were selected from all known genes associated with hereditary retinal dystrophy and then confirmed by Sanger sequencing. Putative pathogenic variants were further validated in available family members and controls. Potential pathogenic mutations were identified in 19 of the 33 probands (57.6%). These mutations were all present in *ABCA4*, but not in the other four STGD-associated genes or in genes responsible for other retinal dystrophies. Of the 19 probands, *ABCA4* mutations were homozygous in one proband and compound heterozygous in 18 probands, involving 28 variants (13 novel and 15 known). Analysis of normal controls and available family members in 12 of the 19 families further support the pathogenicity of these variants. Clinical manifestation of all probands met the diagnostic criteria of STGD. This study provides an overview of a genetic basis for STGD in Chinese patients. Mutations in *ABCA4* are the most common cause of STGD in this cohort. Genetic defects in approximately 42.4% of STGD patients await identification in future studies.

## Introduction

Stargardt Disease (STGD), with a world-wide prevalence of at least 1:10,000 [1], is the most common hereditary macular degeneration in juveniles. It is characterized by macular dystrophy associated with loss of central visual in the first or second decade of life, a “beaten-metal” appearance in the fovea or parafoveal region, yellowish flecks around the macula or in posterior area of the retina, progressive atrophy of the bilateral foveal retinal pigment epithelium, and the “dark choroid” sign on Fundus Fluorescein Angiography (FFA) in 80% of patients [2,3]. STGD is frequently transmitted as an autosomal recessive trait but rarely as an autosomal dominant trait [4,5].

To date, mutations in five genes have been reported to be responsible for classic STGD or “Stargardt-like” disease (S1 Table): fatty acid elongase 4 (*ELOVL4*, MIM: 605512) [6–8], prominin 1 (*PROM1*, MIM: 604365) [5,9], peripherin 2 (*PRPH2*, MIM: 179605) [10,11], Bestrophin 1 (*BEST1*, MIM: 607854) [12] and ATP-binding cassette, sub-family A, member 4 (*ABCA4*, MIM: 601691) [13]. Mutations in these genes are responsible for over half of STGD cases, based on analysis of individual gene or a subset of genes [14,15]. Recently, targeted exome sequencing on 144 or 213 genes detected mutations in 100% (6/6) or 68% (59/88) of families with STGD [15,16], where 13% (11/88) of the families with atypical STGD had mutations in genes other than STGD-associated genes [15]. Additional comprehensive studies are expected to enrich the understanding of the genetic defects responsible for STGD.

In the present study, whole exome sequencing was performed on 33 probands with STGD, and potential pathogenic mutations were detected in 19 of these 33 patients.

## Materials and Methods

### Patients

All 33 probands from unrelated families and available family members were collected from Zhongshan Ophthalmic Center, Sun Yat-sen University. Written informed consent was obtained from participants or their guardians before the collection of their peripheral venous blood and clinical data. This study was approved by the Institutional Review Board of the Zhongshan Ophthalmic Center and was adherent to Declaration of Helsinki. Genomic DNA was extracted from the leukocytes of peripheral venous blood of each participating individual, as well as 96 unrelated healthy individuals [17] and 228 unrelated controls.

### Whole exome sequencing

Genomic DNA from the 33 probands was initially analyzed by whole exome sequencing, as described previously [18]. Briefly, genomic DNA was fragmented and the fragments containing coding regions was captured by an Agilent SureSelect Human All Exon Enrichment Kit V4 array. The exome-enriched DNA fragments were sequenced with an Illumina HiSeq2000 at an average sequencing depth of 125-fold. All sequencing reads were aligned to the consensus sequence (UCSC hg19) with Burrows-Wheeler Aligner (BWA) (http://bio-bwa.sourceforge.net/) and variants were detected with SAMtools (http://samtools.sourceforge.net/). In the current study, variants for all genes related to retinal dystrophy based on RetNet (https://sph.uth.edu/RetNet/home.htm, accessed on August 14, 2014) were selected and then analyzed as follows: 1) exclusion of variants in non-coding regions or synonymous variants without affecting splicing site predicted by BDGP (http://www.fruitfly.org/seq_tools/splice.html); 2) exclusion of variants with minor allele frequency >0.01 determined with 1000 Genomes (http://browser.1000genomes.org/index.html), the Exome Variant Server (http://evs.gs.washington.edu/EVS/), ExAC (http://exac.broadinstitute.org/) and in-house controls; 3) exclusion of single heterozygous variants in genes responsible for recessive diseases; 4) exclusion of variants predicted to be benign by all three online tools (SIFT: http://sift.jcvi.org/www/SIFT_enst_submit.html; PolyPhen-2: http://genetics.bwh.harvard.edu/pph2/; and Proven: http://provean.jcvi.org/genome_submit_2.php?species=human). After the above four steps, the remaining variants were then confirmed by Sanger sequencing and were further evaluated in available family members as well as in controls.

### Sanger Sequencing

For each candidate variant, primers were designed with the online tool Primer3 (http://primer3.ut.ee/) to amplify the genomic fragment harboring the variant (S2 Table). The amplicons were analyzed on an ABI 3130 genetic analyzer (Applied Biosystems, Foster City, CA, USA) with a BigDye Terminator cycle sequencing kit version 3.1. The sequencing results were aligned with consensus sequences from the Genome Bioinformatics database (http://genome.ucsc.edu/) to identify variants by SeqmanII program of DNASTAR Lasergene package (Lasergene version 7.1; DNASTAR, Madison, WI, USA).

## Results

**Fig 1:**
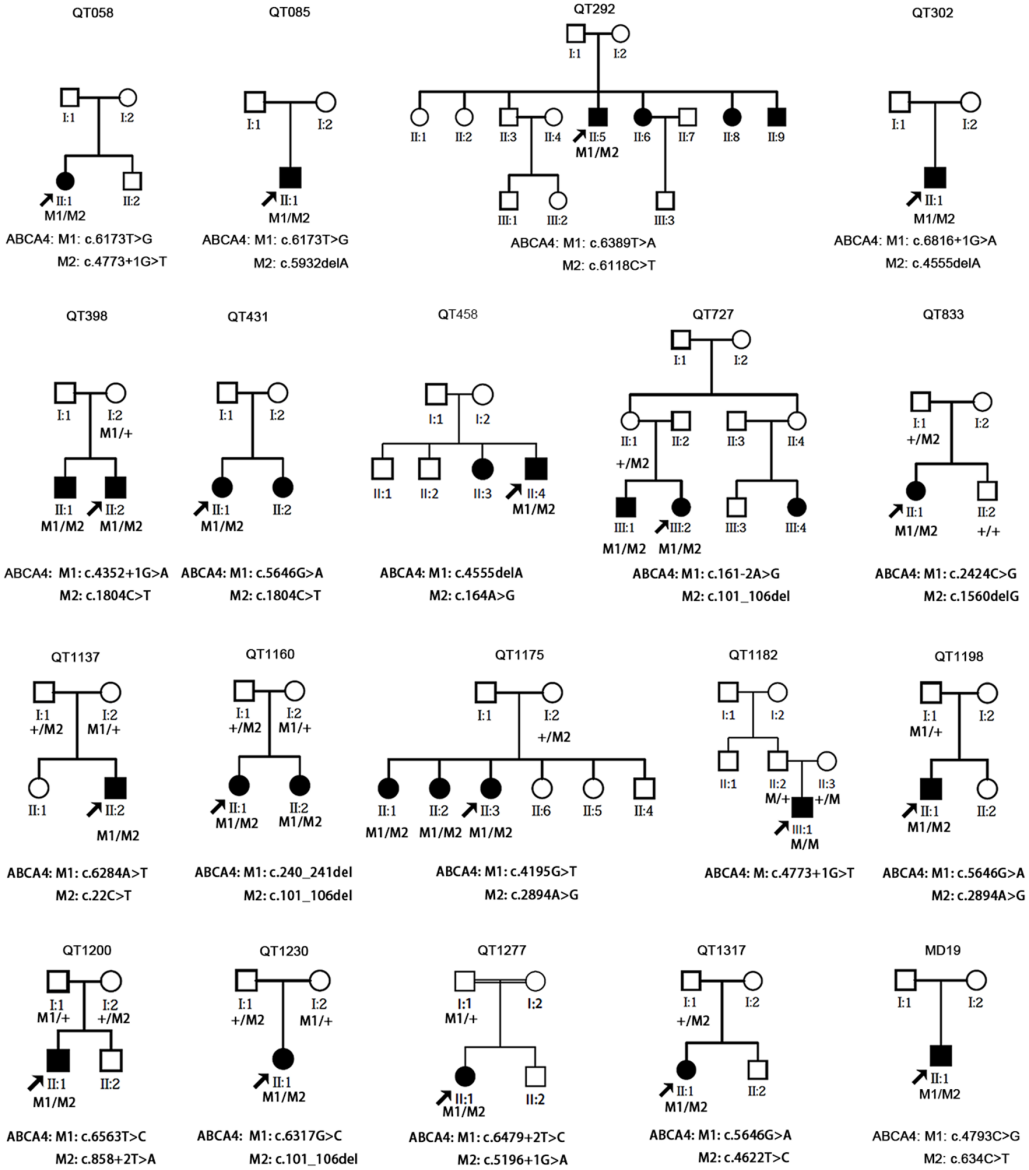
The *ABCA4* mutation and the pedigree. Under each individual, + indicates a wild type allele, and M indicates a mutant allele.

In total, 800 variants were initially detected in 163 genes by whole exome sequencing initially. After multi-bioinformatics filtering and confirmation, 28 potential pathogenic mutations were identified in 19 of the 33 probands (57.6%) (Table 1, S1 Fig). These mutations were all present in *ABCA4*, involving 13 novel and 15 known mutations. *ABCA4* mutations were identified in 19 probands, including one proband with a homozygous mutation and another 18 probands with compound heterozygous mutations. Analysis of controls and available family members in 12 of the 19 families further support the pathogenicity of these variants (Fig 1). No potential mutations were detected in the other four STGD-associated genes (*ELOVL4*, *PROM1*, *PRPH2*, and *BEST1*) or in the 209 genes responsible for other forms of retinal dystrophy. Potential *ABCA4* variants were identified in 8 probands (S3 Table), including 4 probands with two or more heterozygous variants but only one of them was predicted to be pathogenic, and 4 probands with only one heterozygous variant predicted to be pathogenic.

**Table 1:** The *ABCA4* causative variants in 19 Chinese probands with Stargardt disease.

Patient	Nucleotide	Amino Acid	State	Computational Prediction			Allele Frequency in					Reported
ID	Change	Change		P/SS	Proven	SIFT	1000G	EVS	ExAC	NC	RC	
QT058	c.6173T>G	p.L2058R	Het	PrD	D	D	NA	NA	NA	0/192	0/456	Novel
	c.4773+1G>T	Splicing defect	Het	SSA	NA	NA	NA	NA	NA	-	0/456	Pang et al. 2002; Riveiro-Alvarez et al. 2013
QT085	c.6173T>G	p.L2058R	Het	PrD	D	D	NA	NA	NA	0/192	0/456	Novel
	c.5932delA	p.K1978Qfs*13	Het	NA	NA	NA	NA	NA	NA	0/192	0/456	Novel
QT292	c.6389T>A	p.M2130K	Het	PoD	D	D	NA	NA	NA	-	0/456	Yi et al. 2012
	c.6118C>T	p.R2040*	Het	NA	NA	NA	NA	NA	2/121394	0/192	0/456	Baum et al. 2003
QT302	c.6816+1G>A	Splicing defect	Het	SSA	NA	NA	NA	NA	NA	-	0/456	Robert et al. 2014
	c.4555delA	p.T1519Rfs*7	Het	NA	NA	NA	NA	NA	NA	0/192	0/456	Novel
QT398	c.4352+1G>A	Splicing defect	Het	SSA	NA	NA	NA	NA	1/121268	-	0/456	Ernest et al. 2009
	c.1804C>T	p.R602W	Het	PoD	D	D	NA	NA	6/119038	-	2/456	Lewis et al. 1999; Wiszniewski et al. 2005; Heathfield et al. 2013
QT431	c.5646G>A	p.M1882I	Het	PoD	D	D	NA	NA	3/121340	-	0/456	Zernant et al. 2011
	c.1804C>T	p.R602W	Het	B	D	D	NA	NA	6/119038	-	2/456	Lewis et al. 1999; Wiszniewski et al. 2005; Heathfield et al. 2013
QT458	c.4555delA	p.T1519Rfs*7	Het	NA	NA	NA	NA	NA	NA	0/192	0/456	Novel
	c.164A>G	p.H55R	Het	PoD	D	D	NA	NA	NA	-	0/456	Thiadens et al. 2012
QT727	c.161-2A>G	Splicing defect	Het	SSA	NA	NA	NA	NA	NA	0/192	0/456	Novel
	c.101_106del	p.S34_L35del	Het	NA	NA	NA	NA	NA	NA	0/192	0/456	Novel
QT833	c.2424C>G	p.Y808*	Het	NA	NA	NA	NA	NA	NA	-	0/456	Zhou et al. 2014
	c.1560delG	p.V521Sfs*47	Het	NA	NA	NA	NA	NA	NA	0/192	0/456	Novel
QT1137	c.6284A>T	p.D2095V	Het	PrD	D	D	NA	NA	NA	0/192	0/456	Novel
	c.22C>T	p.Q8*	Het	NA	NA	NA	NA	0.0001	NA	0/192	0/456	Novel
QT1160	c.240_241del	p.C81Ffs*17	Het	NA	NA	NA	NA	NA	NA	0/192	0/456	Novel
	c.101_106del	p.S34_L35del	Het	NA	NA	NA	NA	NA	NA	0/192	0/456	Novel
QT1175	c.4195G>T	p.E1399*	Het	NA	NA	NA	NA	NA	2/120596	0/192	0/456	Novel
	c.2894A>G	p.N965S	Het	PrD	D	D	NA	0.0001	21/121302	-	0/456	Allikmets et al. 1997; Shanks et al. 2013; Bertelsen et al. 2014
QT1182	c.4773+1G>T	Splicing defect	Hom	SSA	NA	NA	NA	NA	NA	-	0/456	Pang et al. 2002; Riveiro-Alvarez et al. 2013
QT1198	c.5646G>A	p.M1882I	Het	B	D	D	NA	NA	3/121340	-	0/456	Zernant et al. 2011
	c.2894A>G	p.N965S	Het	PrD	D	D	NA	0.0001	21/121302	-	0/456	Allikmets et al. 1997;Shanks et al. 2013; Bertelsen et al. 2014
QT1200	c.6563T>C	p.F2188S	Het	B	D	D	NA	0.0005	2/121380	-	1/456	Fukui et al. 2002
	c.858+2T>A	Splicing defect	Het	SSA	NA	NA	NA	NA	NA	-	0/456	Zhang et al. 2014
QT1230	c.6317G>C	p.R2106P	Het	PrD	D	D	NA	NA	NA	0/192	0/456	Novel
	c.101_106del	p.S34_L35del	Het	NA	NA	NA	NA	NA	NA	0/192	0/456	Novel
QT1277	c.6479+2T>C	Splicing defect	Het	SSA	NA	NA	NA	NA	NA	0/192	0/456	Novel
	c.5196+1G>A	Splicing defect	Het	SSA	NA	NA	NA	NA	3/49858	-	0/456	Allikmets et al. 1997; Wiszniewski et al. 2005
QT1317	c.5646G>A	p.M1882I	Het	PoD	D	D	NA	NA	3/121340	-	0/456	Zernant et al. 2011
	c.4622T>C	p.L1541P	Het	PrD	D	D	NA	NA	NA	0/192	0/456	Novel
MD19	c.4793C>G	p.A1598G	het	PoD	D	N	NA	0.0001	NA	-	0/456	Maugeri et al. 2000; Cideciyan et al. 2009; Burke et al. 2010
	c.634C>T	p.R212C	het	D	D	D	NA	0.0002	14/120056	-	0/456	Gerber et al.1998; Thiadens et al. 2012

The following abbreviations are used: P/SS, Polyphen-2/Splice Site Prediction; 1000G, 1000 Genomes; EVS, Exome Variant Server; ExAC, Exome Aggregation Consortium; Het, heterozygous; Hom, homozygous; NC, normal control; RC, relative control; PrD, probably damaging; PoD, possibly damaging; B, benign; SSA, splicing site abolished; N, neutral; D, damaging; and NA, not applicable;-, not done.

**Fig 2:**
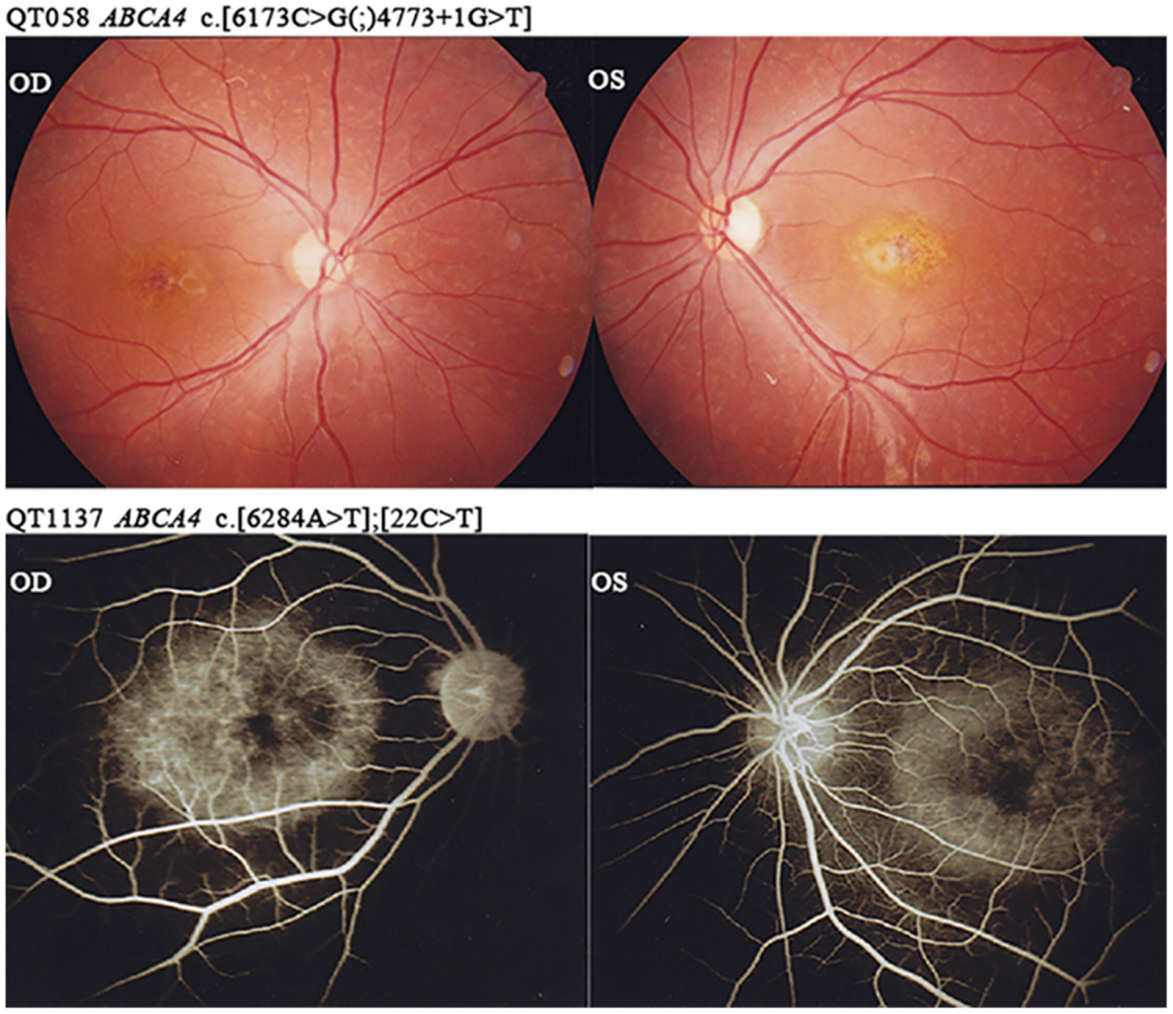
Fundus photographs and Fundus Fluorescein Angiography from QT058 and QT1137 respectively. The *ABCA4* mutations were listed above each photograph identified in this study. OD and OS represent right and left eyes, respectively. Fundus photographs showed yellowish flecks in all posterior of the retina and macular atrophy. FFA showed “dark choroid” sign. More clinical information about these patients is listed in Table 2.

Clinical data of the 19 probands with ABCA4 mutations were listed in Table 2. Most of the probands had complaint of poor vision in their first decades. Ophthalmoscope examination of the fundus revealed one or more of typical clinic features, including a “beaten-metal” appearance in the foveal or parafoveal macula, yellowish flecks around the macula or in posterior retina, retinal pigment epithelium (RPE) atrophy and pigment disorder to different degrees, and a “dark” or “silent” choroid on Fundus Fluorescein Angiography (FFA) (Fig 2).

**Table 2:** Clinical features of Stargardt disease probands with the *ABCA4* mutations identified in this study.

Patient	Variations	Gender	Age at		First	BCVA	Fundus	Dark Choroid
ID			exam	onset	symptom	OD/OS	examination	FFA
QT058	c.[6173T>G(;)4773+1G>T]	F	18	8	PV	0.1/0.2	BMS; PD,YF	Present
QT085	c.[6173T>G(;)5932delA]	M	9	7	PV	0.1/0.1	BMS; PD	NA
QT292	c.[6389T>A(;)6118C>T]	M	25	NA	PV	0.05/0.1	BMS; PD,YF	Present
QT302	c.[6816+1G>A(;)4555delA]	M	17	EC	PV	0.3/0.2	PD	NA
QT398	c.[4352+1G>A];[1804C>T]	M	8	EC	PV	0.06/0.08	BMS; PD	NA
QT431	c.[5646G>A(;)1804C>T]	F	12	10	PV	0.2/0.2	PD	NA
QT458	c.[4555delA(;)164A>G]	F	20	EC	PV	0.04/0.04	PD	NA
QT727	c.[161-2A>G];[101_106del]	F	15	11	PV	0.1/0.05	BMS; PD; YF	NA
QT833	c.[2424C>G];[1560delG]	F	9	NA	PV	0.1/0.07	NA	NA
QT1137	c.[6284A>T];[22C>T]	M	7.5	7	PV	0.07/0.1	PD	Present
QT1160	c.[240_241del];[101_106del]	F	11	6	PV	0.1/0.1	BMS; PD	Present
QT1175	c.[4195G>T];[2894A>G]	F	11	8	PV	0.1/0.1	BMS; PD	NA
QT1182	c.[4773+1G>T];[4773+1G>T]	M	7	NA	NA	NA/NA	PD	NA
QT1198	c.[5646G>A];[2894A>G]	M	8	8	PV	0.2/0.15	BMS; PD	NA
QT1200	c.[6563T>C];[858+2T>A]	M	26	20	PV	0.1/0.1	BMS; PD	Not Present
QT1230	c.[6317G>C];[101_106del]	F	8.5	8	PV	0.2/0.3	BMS	Present
QT1277	c.[6479+2T>C];[5196+1G>A]	F	7	6	PV	0.1/0.1	PD	NA
QT1317	c.[5646G>A];[4622T>C]	F	10	EC	PV	NA/NA	NA	NA
MD19	c.[4793C>G);(634C>T]	M	12	NA	NA	NA/NA	BMS;PD	NA

The following abbreviations are used: M, male; F, female; EC, early childhood; NA, not available; OD, right eye; OS, left eye; PV, poor vision; BCVA, Best corrected visual acuity; PD, pigment disorder; BMS, beaten-metal sign; FFA, Fundus Fluorescein Angiography; YF, yellowish fleck.

## Discussion

In this study, we identified 28 mutations of *ABCA4* in 19 of 33 (57.6%) unrelated probands with STGD. No potential pathogenic variations were detected in other four STGD-related genes and 209 genes responsible for other forms of retinal dystrophies listed in RetNet (S3 Table).

Previously, different sequencing technologies have been used to detect genetic defects of STGD patients in different populations [14–16,19–26]. Mutations in *ABCA4* are the most common cause of STGD in previous studies [15,19,21,25]. Similarly, mutations *ABCA4* have been detected in 57.6% STGD probands in our Chinese cohort. Several specific alleles have been reported in different populations [19–21], but no specific alleles were found in Chinese population in the present study due to the limited number of patients. The 15 known *ABCA4* mutations were also found in the present study. Different patients with known mutations have different phenotypes, previously reports revealed that c.4773+1G>T and c.5196+1G>A associated with AMD (Age-related Macular Degeneration) and c.164A>G (p.H55R) associated with for cone-rod dystrophy [27–29], while homozygous c.4773+1G>T and compound heterozygous c.5196+1G>A and c.164A>G were associate with STGD in our study. Such varied phenotypes may be determined by the second mutation [30]. Three novel *ABCA4* mutations were identified in three small families (QT085, QT302, and QT358) where family members was not available for analysis. These mutations occur in different protein domains, including K1978Qfs*13 and L2058R in cytosolic nucleotide binding domain 1 and T1519Rfs*7 in exocytoplasmic domains 2.

Except for *ABCA4* mutations in 19 of 33 probands, potential mutations have not been detected in the other 14 probands after analysis of other 213 genes in which mutations were responsible for different forms of retinal dystrophy based on comprehensive whole exome analysis. No mutations identified in other genes except for *ABCA4* may partly because of the limited number of probands analyzed. A previous study [15] also reported no pathogenic mutations in about 30% of patients with STGD. This suggests that as yet unidentified genes may also contribute to STGD. Therefore, further bioinformatics analysis on data resulted from whole exome sequencing may lead to find new genes responsible for STGD in the remaining probands without mutations. In addition, mutations in the noncoding regions may be missed by whole exome sequencing. Additional analysis using different strategies are expected to find such mutations, especially for those probands with single pathogenic variants in recessive genes.

## Supporting Information

S1 Fig Sequence chromatography. 38 sequence changes that were detected in 19 probands with Stargardt disease are shown (left column) and compared with corresponding normal sequences (right column).
(PDF)

S1 Table Genomic information of the five known genes responsible for STGD. (XLSX)

S2 Table Primers used in Sanger sequencing. (XLSX)

S3 Table Potential pathogenic *ABCA4* variants in other 8 probands with Stargardt disease. (XLSX)
