# Supplementary material for: Identification of Genetic Defects in 33 Probands with Stargardt Disease by WES-Based Bioinformatics Gene Panel Analysis
Source: PLoS One. 2015 Jul 10;10(7):e0132635. doi: 10.1371/journal.pone.0132635 (PMC4498695; doi:10.1371/journal.pone.0132635)
Supplement: S1 Fig — 38 sequence changes that were detected in 19 probands with Stargardt disease are shown (left column) and compared with corresponding normal sequences (right column). (PDF) [file pone.0132635.s001.pdf]

Supplementary Fig. S1. Sequence chromatography. 38 sequence changes that were detected in 19 probands with Stargardt disease are shown (left column) and compared with corresponding normal sequences (right column).

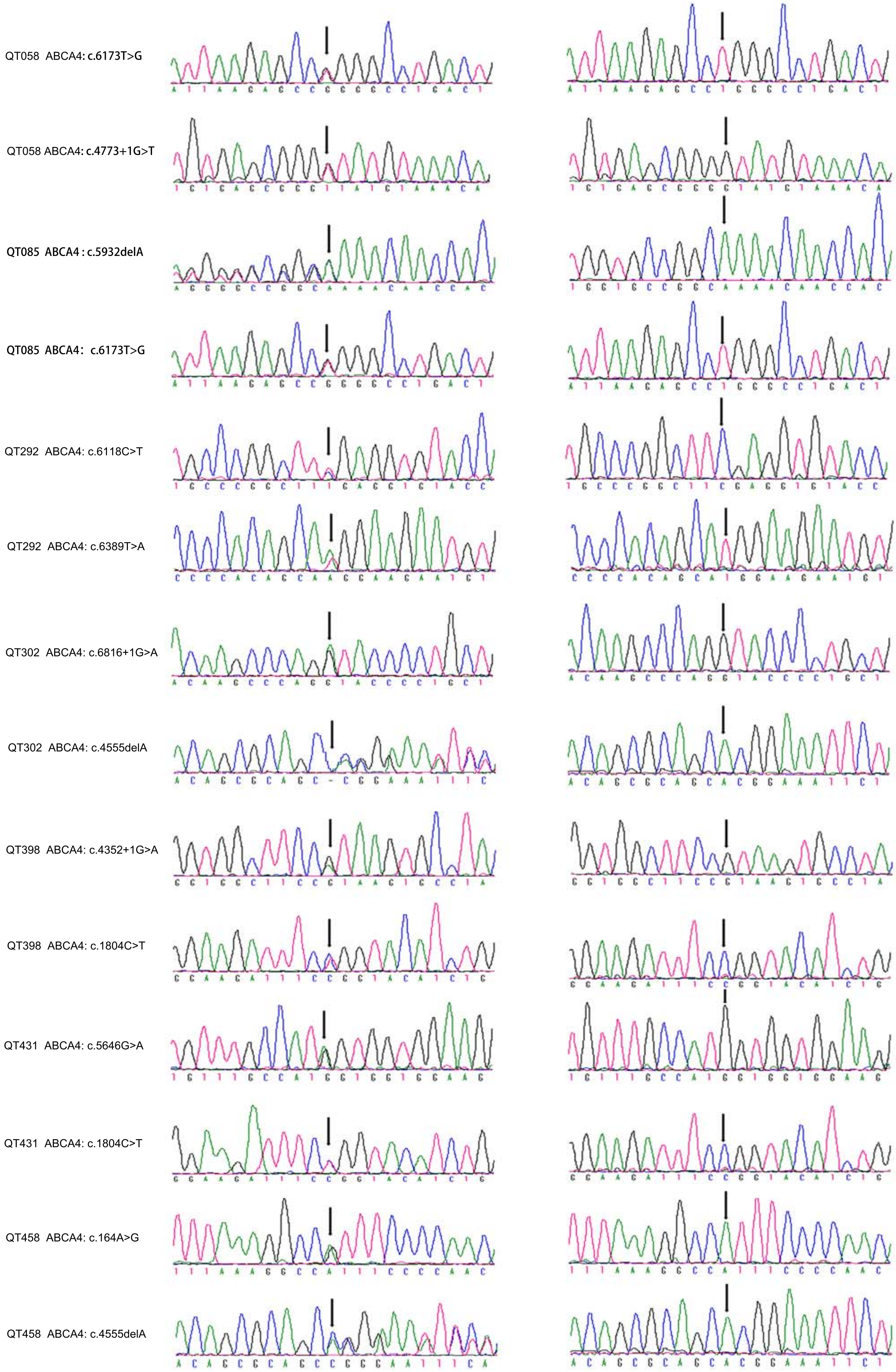

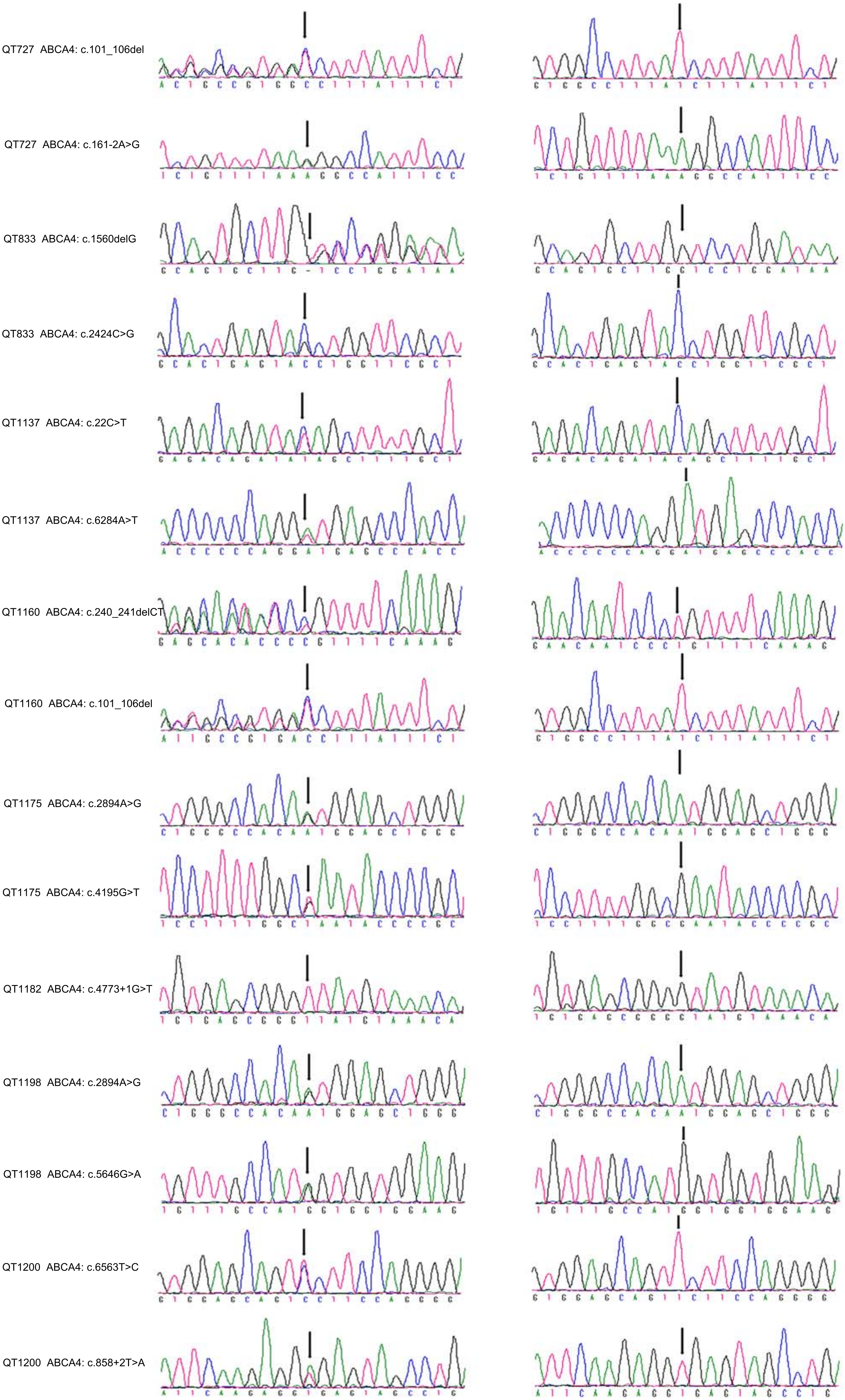
QT727 ABCA4: c.101_106del
QT727 ABCA4: c.161-2A>G
QT833 ABCA4: c.1560delG
QT833 ABCA4: c.2424C>G
QT1137 ABCA4: c.22C>T
QT1137 ABCA4: c.6284A>T
QT1160 ABCA4: c.240_241delCT
QT1160 ABCA4: c.101_106del
QT1175 ABCA4: c.2894A>G
QT1175 ABCA4: c.4195G>T
QT1182 ABCA4: c.4773+1G>T
QT1198 ABCA4: c.2894A>G
QT1198 ABCA4: c.5646G>A
QT1200 ABCA4: c.6563T>C
QT1200 ABCA4: c.858+2T>A

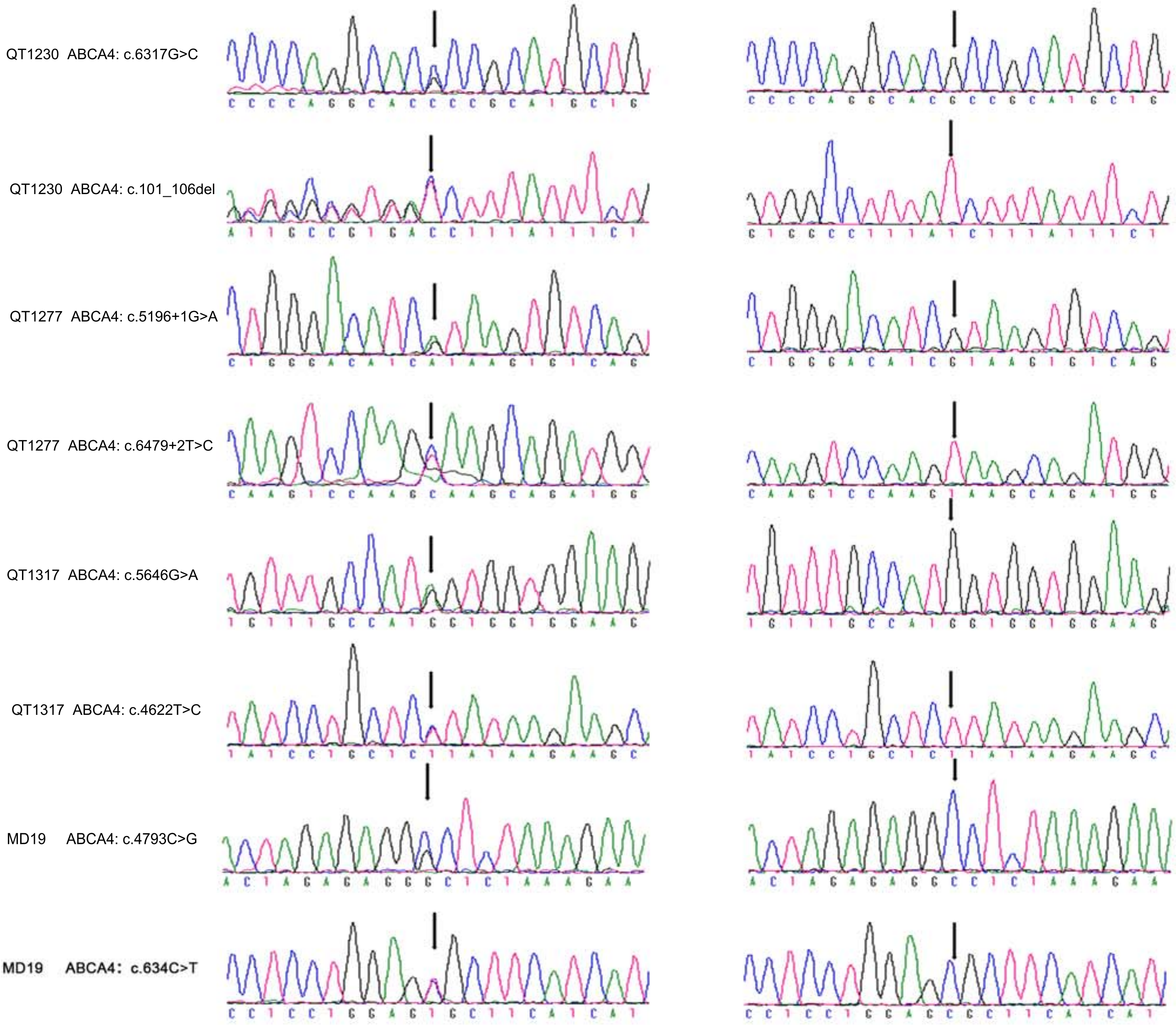
QT1230 ABCA4: c.6317G>C
QT1230 ABCA4: c.101_106del
QT1277 ABCA4: c.5196+1G>A
QT1277 ABCA4: c.6479+2T>C
QT1317 ABCA4: c.5646G>A
QT1317 ABCA4: c.4622T>C
MD19 ABCA4: c.4793C>G
MD19 ABCA4：c.634C>T
